# Sex-Specific Downregulation of CDK5RAP3 Exacerbates ER Stress-Mediated Inflammation and Apoptosis in CCl_4_-Induced Acute Liver Injury

**DOI:** 10.3390/genes17010073

**Published:** 2026-01-08

**Authors:** Jian Ruan, Qianyi Dong, Fangling Xu, Yufan Jin, Yuhong Yang, Jun Li, Yafei Cai

**Affiliations:** 1 Anhui Provincial Key Lab of the Conservation and Exploitation of Biological Resources, College of Life Science, Anhui Normal University, Wuhu 241000, China; 2 College of Animal Science and Technology, Nanjing Agricultural University, Nanjing 210095, China

**Keywords:** CDK5RAP3, CCl_4_, liver, sex

## Abstract

Background/Objectives: Sex-specific differences in the mechanisms of acute liver injury remain poorly understood. CDK5 regulatory subunit-associated protein 3 (CDK5RAP3) is crucial for liver development and endoplasmic reticulum (ER) homeostasis. This study aimed to investigate sex-dependent changes in CDK5RAP3 expression in a carbon tetrachloride (CCl_4_)-induced acute liver injury model and to explore the mechanisms underlying differential susceptibility between males and females. Methods: Acute liver injury was induced in male and female mice by CCl_4_ administration. Liver injury was evaluated by serum biochemical parameters and histopathological analysis. CDK5RAP3 expression, inflammatory cytokines, and ER stress-related apoptotic markers were assessed. Hepatocyte apoptosis was examined by TUNEL staining. In addition, CDK5RAP3 was conditionally deleted in mouse embryonic fibroblasts (MEFs) using 4-hydroxytamoxifen to assess its direct role in regulating inflammatory and apoptotic responses in vitro. Results: CCl_4_ exposure caused liver injury in both sexes, with male mice showing more severe biochemical and histological damage. CDK5RAP3 expression was significantly reduced after CCl_4_ treatment, particularly in males. Inflammatory mediators and ER stress-associated apoptotic markers were upregulated, accompanied by increased hepatocyte apoptosis. A similar enhancement of inflammatory and apoptotic signaling was observed in CDK5RAP3-deficient MEFs. Conclusions: Downregulation of CDK5RAP3 is associated with ER stress, inflammation, and apoptosis, contributing to increased susceptibility of male mice to acute liver injury. These findings provide insight into sex-specific mechanisms of hepatic injury and highlight CDK5RAP3 as a potential therapeutic target.

## 1. Introduction

Cyclin-dependent kinase 5 regulatory subunit-associated protein 3 (CDK5RAP3), also known as LZAP or C53, is highly conserved among species and functions as a binding protein partner of the cyclin-dependent kinase 5 (CDK5) activator. CDK5RAP3 is expressed in various mammalian organs, with the highest expression in the liver [1]. In addition to interacting with proteins in the UFM1 coupling system such as UFBP1, UFM1, and UFL1 [2,3,4,5], CDK5RAP3 also interacts with multiple signaling molecules, including RelA, ARF, Chk1, RPL26, PAK4, P38, and STAT3, to regulate cell cycle progression, cell survival, cell adhesion and invasion, tumorigenesis, and metastasis [6,7,8,9,10]. Studies have shown that CDK5RAP3 is a crucial factor in the development and proper function of the mammalian liver. It regulates critical processes, including liver cell proliferation and differentiation, UFMylation, and ER homeostasis [11]. In addition, a significant decrease in CDK5RAP3 expression has been reported in both liver tumor tissues and hepatocellular carcinoma cell lines [12]. Therefore, CDK5RAP3 may be closely associated with the onset and progression of liver diseases, as well as sex-related differences in liver injury severity.

The liver is a highly sexually dimorphic organ, with approximately 72% of hepatic genes exhibiting sex-biased expression. This dimorphism contributes to differences in susceptibility, progression, and outcomes of numerous acute and chronic liver diseases between females and males [13]. Male-biased susceptibility to liver disease is a well- recognized phenomenon in mammals, yet the underlying molecular mechanisms remain poorly understood [14]. Carbon tetrachloride (CCl_4_)-induced acute liver injury is a classic experimental model for mimicking human liver diseases [15,16]. In a CCl_4_-induced acute liver injury model in rats, female livers exhibited a significantly weaker fibrotic response compared with male livers, and estradiol played a key inhibitory role in liver fibrosis development [17].

In this study, we systematically compared liver function parameters and histopathological alterations among experimental groups to characterize sex differences in CCl_4_-induced acute liver injury in mice. We further investigated the expression profile of CDK5RAP3 in this model and explored its potential involvement in sex-dependent susceptibility to acute liver injury. In addition, we analyzed the associations between CDK5RAP3 expression and key inflammatory and apoptotic mediators, including TNF-α, IL-1β, NLRP3, BAX, and CHOP. Our findings suggest that sex-related differences in liver injury severity may be associated with the downregulation of CDK5RAP3. Loss of CDK5RAP3 enhances inflammatory signaling and apoptotic pathways, disrupts ER homeostasis, and ultimately promotes hepatocyte apoptosis.

## 2. Materials and Methods

### 2.1. Animals

A total of 16 KM mice (8 males and 8 females, 6–8 weeks old, weighing 20–25 g) were maintained under specific pathogen-free (SPF) housing conditions with standard husbandry and ad libitum access to food and water (25 ± 1 °C, humidity 40–80%). All animal procedures were conducted in accordance with the Animal Management Regulations issued by the National Science and Technology Commission of the People’s Republic of China and were approved by the Animal Ethics Committee of Anhui Normal University (Approval No. AHNU-ET2025055).

### 2.2. Animal Experiments and Study Participants

CCl_4_ (Shanghai Macklin Biochemical Co., Ltd., Shanghai, China) was dissolved in olive oil to prepare a 15% (*v*/*v*) CCl_4_ solution, which was administered to mice by a single intraperitoneal injection at a dose of 0.1 mL/10 g body weight to induce acute liver injury. The acute liver injury model was established 24 h after injection. Control mice received an intraperitoneal injection of the same volume of olive oil. Each group included female control, female model, male control, and male model groups.

### 2.3. Determination of Biochemical Indexes and Its Methods

After 24 h, mice were anesthetized with 1% pentobarbital sodium, and blood samples were collected from the retro-orbital plexus. Following centrifugation, serum samples were analyzed by Servicebio Co. (Hefei, China) using a fully automated biochemical analyzer to determine the levels of alanine aminotransferase (ALT), aspartate aminotransferase (AST), total bilirubin (TBIL), direct bilirubin (DBIL), and albumin.

### 2.4. Histopathological Analysis and TUNEL

After euthanasia, liver tissues were collected from the same anatomical region of the right hepatic lobe and trimmed into 1 × 1 cm blocks. Tissues were fixed in 4% paraformaldehyde, embedded in paraffin, and sectioned into 5-μm-thick slices. Hematoxylin and eosin (H&E) staining was performed to evaluate histopathological changes under a light microscope. TUNEL staining was conducted according to the manufacturer’s instructions using a TUNEL apoptosis detection kit (CY3; BOSTER, Wuhan, China) to assess hepatocyte apoptosis.

### 2.5. Quantitative Real-Time PCR

The liver tissue samples were treated with Trizol (Invitrogen, Carlsbad, CA, USA, 15596026CN) to extract total RNA, and the RNA concentration was quantified using a multi-functional microplate reader. The RNA was then reverse-transcribed into cDNA using a reverse transcription kit (Tiangen Biochemical Technology, Beijing, China). The cDNA was subjected to Q-PCR on a Bio-Rad fluorescence quantitative PCR instrument using the following amplification conditions: pre-denaturation at 95 °C for 5 min, followed by 50 cycles of denaturation at 95 °C for 30 s, annealing at 55 °C for 30 s, and extension at 72 °C for 20 s. Primer sequences specifically designed for the amplification of target transcripts are detailed in Table 1. This approach ensured precise and reliable quantification of gene expression.

### 2.6. Western Blot Analysis

The total protein of liver tissues or MEFs cell was extracted using RIPA reagent (Beyotime, Shanghai, China, P0013E), and the concentration was determined by the BCA method (Beyotime, P0010S). The protein was denatured, and then the protein lysate was electrophoresed using SDS-PAGE and transferred to a PVDF membrane. The membrane was incubated overnight with primary antibodies of β-actin (Solarbio, Beijing, China, K200058M), CDK5RAP3 (Abcam, Cambridge, MA, USA, ab157203), NLRP3 (Proteintech, Wuhan, China, 19771-1-AP), BAX (Solarbio, K008076P), and CHOP (Solarbio, K107245P). The next day, the primary antibody dilution was removed, and the membrane was washed three times with TBST. Then the membrane was incubated with goat anti-mouse or goat anti-rabbit for 1 h, washed three times with TBST, and then photographed and analyzed with an enhanced chemiluminescent solution detection system. The bands obtained were quantitatively analyzed by the Image J 1.54g (National Institutes of Health, Bethesda, MD, USA) image analysis software.

### 2.7. Cell Culture

Immortalized mouse embryonic fibroblasts (MEFs) were generated by using CDK5RAP3 F/F: CAG-CreERT2 mice as previously reported [8]. For CDK5RAP3 knockdown generation, MEFs were treated with 4-OHT (4-Hydroxytamoxifen, 8 μM, H7904 or H6278, Sigma, St. Louis, MO, USA) for 72 h, with EtOH (0.1% ethanol) as control group. All cells, growing in basic DMEM (Gibco, Thermo Scientific, Waltham, MA, USA) supplemented with 10% fetal bovine serum (Gibco, Thermo Scientific, Waltham, MA, USA) and 1% penicillin/streptomycin (Solarbio, Beijing, China), were maintained at 37 °C under 5% CO_2_ atmosphere.

### 2.8. Statistical Analysis

Statistical analyses were performed using GraphPad Prism version 8.0 (GraphPad Software, San Diego, CA, USA). For animal experiments, data were analyzed using two-way analysis of variance (ANOVA) with treatment and sex as independent factors, and the interaction between treatment and sex was assessed. For in vitro cell experiments involving two-group comparisons, data were analyzed using the Mann–Whitney U test. Data are presented as the mean ± standard error of the mean (SEM). A value of *p* < 0.05 was considered statistically significant.

## 3. Results

### 3.1. Serum Biochemical Indicators in CCl_4_-Induced Liver Injury Mice

**Figure 1:**
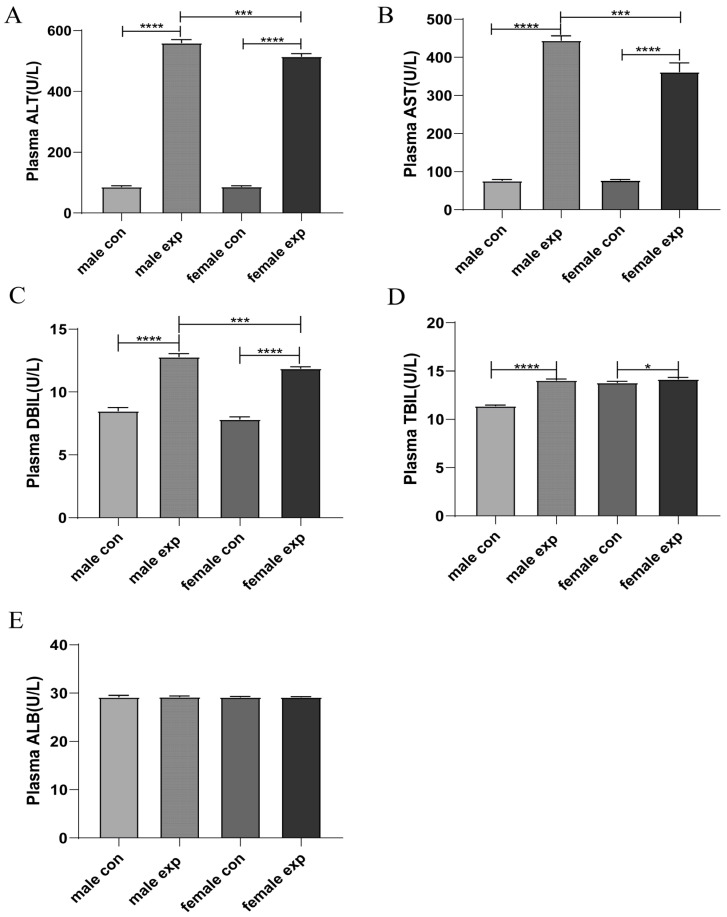
Serum biochemical parameters in CCl_4_-induced acute liver injury. Serum ALT, AST, DBIL, TBIL and ALB levels were measured 24 h after CCl_4_ or olive oil administration in male and female mice (**A**–**E**). Data are presented as mean ± SEM (n = 4). * *p* < 0.05; *** *p* < 0.001; **** *p* < 0.0001. male con, male control group; male exp, CCl_4_-induced male acute liver injury experimental group; female con, female control group; female exp, CCl_4_-induced female acute liver injury experimental group.

As shown in Figure 1, 24 h after administration of CCl_4_ or olive oil, serum AST, ALT, TBIL, and DBIL levels were markedly elevated in mice treated with CCl_4_ compared with the control group (*p* < 0.05), confirming the successful establishment of the acute liver injury model. In contrast, serum ALB levels did not differ significantly between control and CCl_4_-treated mice. Moreover, male mice in the CCl_4_-treated group exhibited significantly higher AST, ALT, and DBIL levels than their female counterparts (*p* < 0.05). Notably, TBIL levels in female mice did not show a significant increase compared with male mice, consistent with the overall milder degree of liver injury observed in females.

### 3.2. Gender Differences in the Histopathology of Liver Tissues

**Figure 2:**
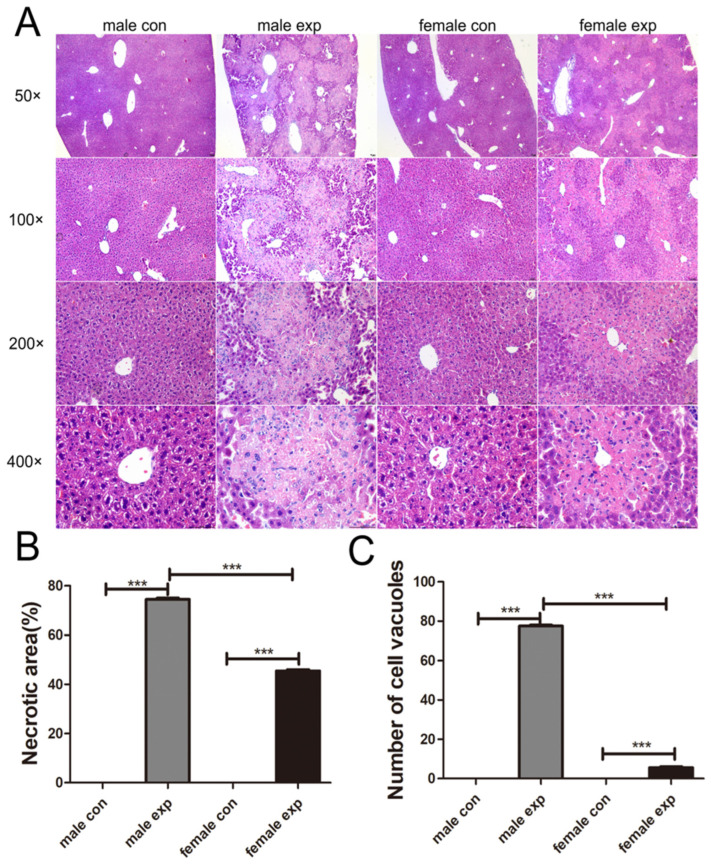
Histopathological assessment of liver injury. (**A**) Representative H&E-stained liver sections from each group (original magnification: 50×, 100×, 200×, 400×); (**B**) Statistical analysis of Necrotic area of liver tissue; (**C**) Statistical analysis of cell vacuole number in liver tissue; Five random fields per section were analyzed. Data are presented as mean ± SEM (n = 4) (*** *p* < 0.001).

We next evaluated liver injury using HE staining. As shown in Figure 2, the control groups exhibited intact and clear well-defined hepatocyte morphology and hepatic lobule architecture, with no evidence of necrosis or abnormalities in the liver sinusoids or blood vessels. In contrast, the model groups displayed marked structural disruption, characterized by blurred lobular architecture, central coagulative necrosis, and extensive hepatocellular degeneration, necrosis, and inflammatory cell infiltration. The hepatocyte cytoplasm appeared loosened, vacuolated, and lightly stained. Notably, male mice in the model group demonstrated varying degrees of fatty degeneration with prominent large lipid droplets, whereas female mice showed minimal or no fatty changes. Inflammatory changes were more pronounced in male mice, consistent with the greater extent of hepatocellular injury observed in this group. Quantitative analysis revealed that the area of liver injury and the number of vacuolated hepatocytes were significantly higher in male mice than in female mice (*p* < 0.001). These results indicate a clear gender difference in the CCl_4_-induced acute liver injury model, with males exhibiting more severe hepatic damage.

### 3.3. Changes in CDK5RAP3 Expression in the Acute Liver Injury Model Group

**Figure 3:**
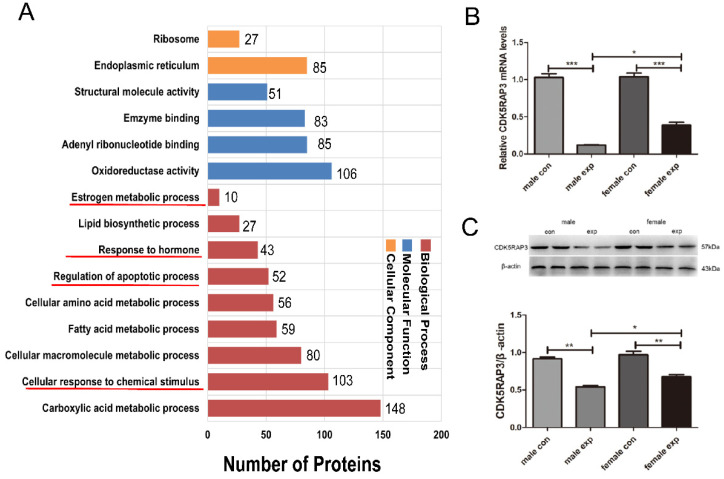
CDK5RAP3 expression in CCl_4_-induced acute liver injury. (**A**) GO annotation of CDK5RAP3-related proteins identified by LC-MS, red underlines highlight selected biological processes of special relevance to the study focus; (**B**) The mRNA expressions of CDK5RAP3 gene were detected in CCl_4_-induced acute liver injury in mice; (**C**) CDK5RAP3 protein expression and densitometric quantification normalized to β-actin; Data are presented as the means ± SEM (n = 4). * *p* < 0.05; ** *p* < 0.01; *** *p* < 0.001).

To investigate the role of CDK5RAP3 in the liver, co-immunoprecipitation (Co-IP) followed by LC–MS analysis was conducted to identify CDK5RAP3-interacting proteins (Table S1). Enrichment analysis revealed that 103 associated proteins were involved in cellular responses to chemical stimuli, 52 in the regulation of apoptotic processes, 43 in responses to hormones, and 10 in estrogen metabolic processes (Figure 3A). Given these findings, we hypothesized that CDK5RAP3 expression might differ between sexes in the CCl_4_-induced acute liver injury model. Therefore, qRT-PCR and Western blot analyses were performed to assess changes in CDK5RAP3 mRNA and protein expression. Compared with the control group, CDK5RAP3 expression at both the mRNA and protein levels was significantly reduced in the model group (*p* < 0.05). Moreover, under control conditions, CDK5RAP3 mRNA and protein levels did not differ significantly between male and female mice. In contrast, following CCl_4_ treatment, CDK5RAP3 expression was significantly reduced in male mice compared with female mice (*p* < 0.05), as shown in Figure 3B,C.

### 3.4. Gender Differences in the Inflammatory Response to CCl_4_ Injection

**Figure 4:**
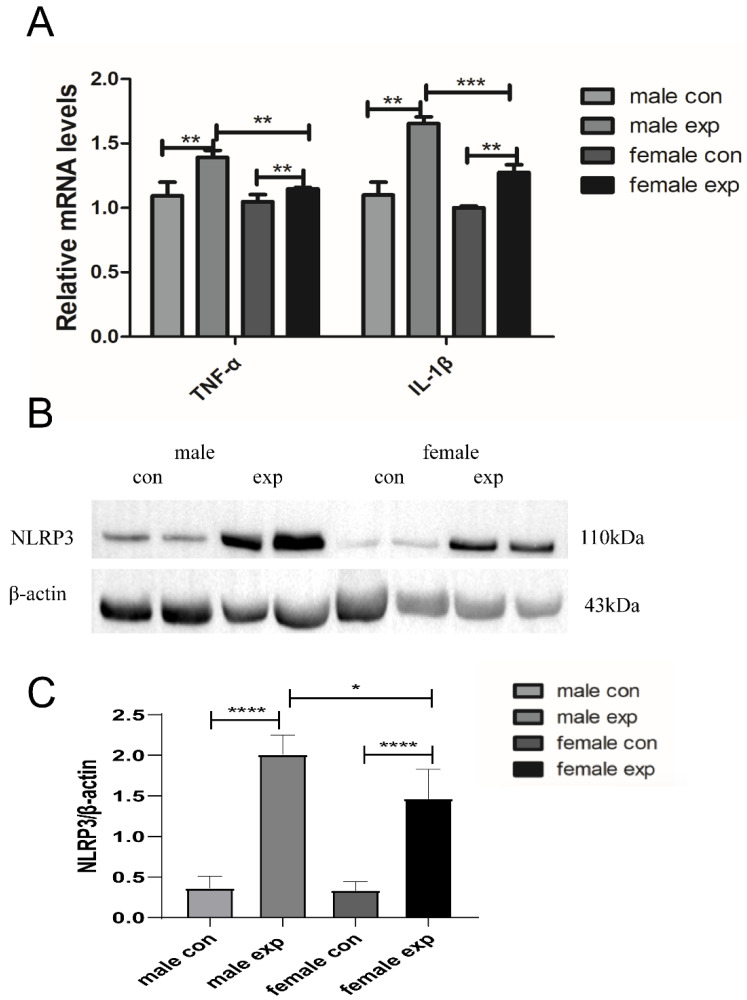
Sex difference inflammatory response in CCl_4_-induced acute liver injury in mice. (**A**) The mRNA expressions of TNF-α and IL-1 β were detected in CCl_4_-induced acute liver injury in mice; (**B**) The level of NLRP3 protein expression in the mice was detected by Western blot; (**C**) Densitometric quantification of NLRP3 normalized to β-actin; Data are presented as mean ± SEM (n = 4) * *p* < 0.05; ** *p* < 0.01; *** *p* < 0.001, ****p < 0.0001.

It is well known that CCl_4_-induced acute liver injury is closely associated with inflammation. To examine potential sex-dependent differences in the inflammatory response triggered by CCl_4_, we measured the expression levels of the inflammation-related factors TNF-α and IL-1β using qRT-PCR. As shown in Figure 4A, both inflammatory markers were significantly upregulated in the model group compared with the control group (*p* < 0.01). Furthermore, under control conditions, female mice exhibited lower basal expression levels of TNF-α and IL-1β compared with males. Following CCl_4_ treatment, the expression of both cytokines was significantly increased in both sexes, with male mice showing a greater magnitude of induction (*p* < 0.01). In Figure 4B,C, Western blot analysis was performed to assess NLRP3 protein expression. Compared with the control group, NLRP3 protein levels were significantly elevated in the model group (*p* < 0.01). Moreover, NLRP3 expression was higher in male model mice than in female model mice (*p* < 0.05). These results indicate that CCl_4_-induced acute liver injury elicits a strong inflammatory response and that this response exhibits clear sex differences, with male mice displaying more pronounced inflammatory activation than female mice.

### 3.5. Gender Difference in CCl_4_-Induced Liver Injury-Related Apoptosis in Mice

**Figure 5:**
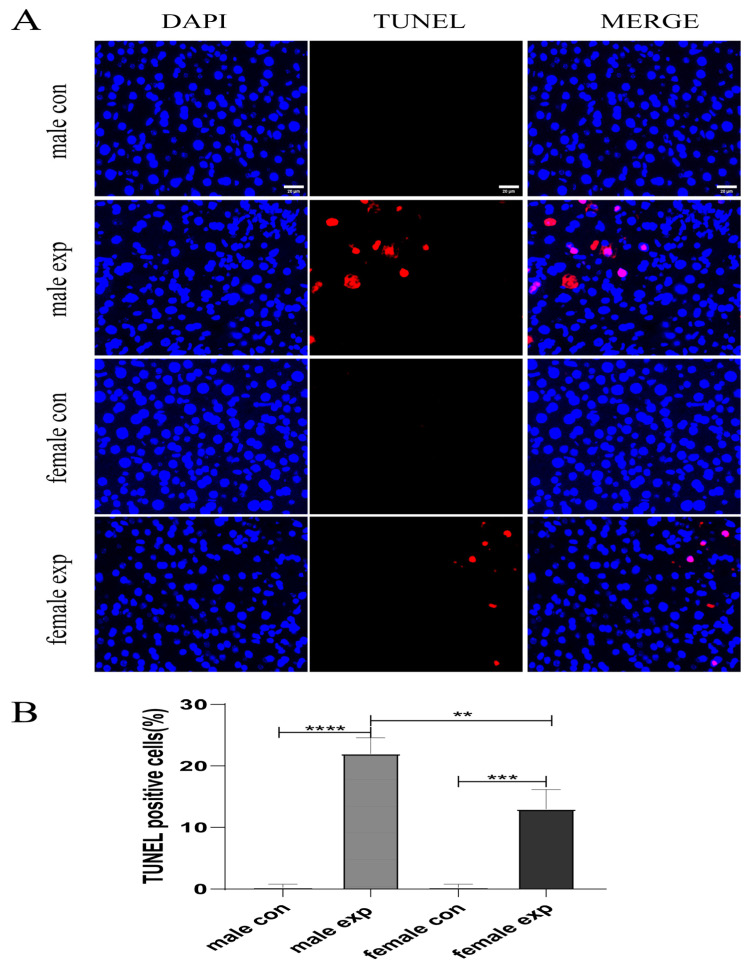
Sex difference in hepatocyte apoptosis in CCl_4_-induced acute liver injury in mice. (**A**) Representative TUNEL-stained liver sections 24 h after CCl_4_ administration (red; original magnification ×200; scale bar = 50 μm); (**B**) Apoptotic cells were quantified by TUNEL staining; Five random fields per section were analyzed. Data are presented as mean ± SEM (n = 4). ** *p* < 0.01; *** *p* < 0.001, **** *p* < 0.0001.

**Figure 6:**
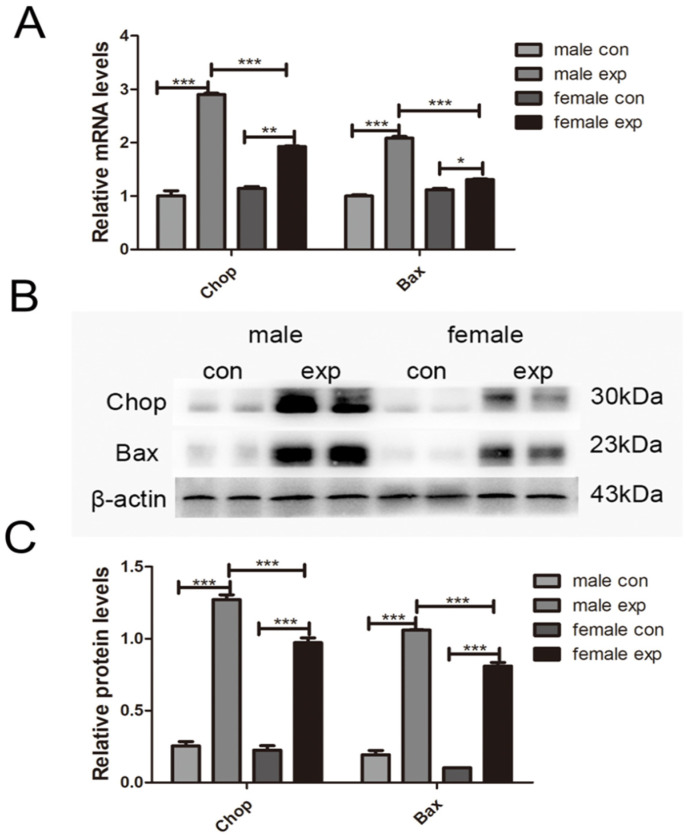
Sex differences in the mRNA and protein expressions of Chop and Bax in CCl_4_-induced acute liver injury in mice. (**A**) The mRNA expressions of Chop and Bax was detected by QRT-PCR; (**B**) The level of Chop and Bax protein expression in the mice was detected by Western blot; (**C**) Densitometric quantification normalized to β-actin; Data are presented as mean ± SEM (n = 4) (* *p* < 0.05; ** *p* < 0.01; *** *p* < 0.001).

Endoplasmic reticulum (ER) stress plays a crucial role in maintaining cellular homeostasis, and its dysregulation is implicated in many liver diseases. Hepatocyte apoptosis is a key event in the early stages of acute liver injury. Therefore, we evaluated sex-related differences in hepatocyte apoptotic in the acute liver injury model using a TUNEL assay. As shown in Figure 5, the number of apoptotic cells in liver tissues was significantly higher in male model mice than in female model mice. This observation was further supported by qRT-PCR and Western blot analyses, which demonstrated marked upregulation of the apoptosis-related genes Chop and Bax in the model group, with particularly pronounced increases in male model mice (*p* < 0.01) (Figure 6). These findings suggest that hepatocytes undergo apoptosis following acute liver injury and that sex-dependent differences in apoptosis-related marker expression are associated with the greater severity of liver injury observed in male mice.

### 3.6. Loss of CDK5RAP3 Is Associated with Inflammatory Responses and Apoptosis

**Figure 7:**
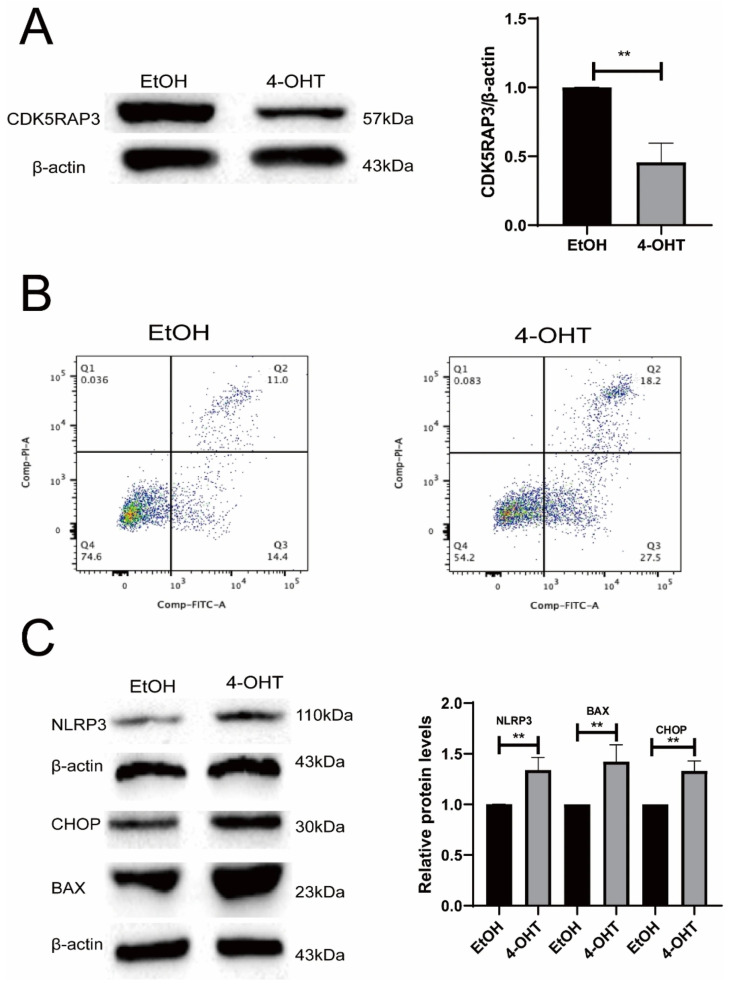
CDK5RAP3 knockdown-associated inflammatory and apoptotic responses in MEFs. (**A**) Western blot validation of CDK5RAP3 protein expression in MEFs after 72 h treatment with 4-OHT (8 μM); (**B**) Apoptotic cell percentages detected by Annexin V/PI flow cytometry, each dot corresponds to a single cell, and the pseudocolor scale represents cell density (blue to red, low to high); (**C**) Densitometric quantification of NLRP3, CHOP, and BAX normalized to β-actin; Data are expressed as mean ± SEM from three independent experiments (n = 3). ** *p* < 0.01.

To elucidate the regulatory mechanisms underlying CDK5RAP3 deficiency in inflammatory and apoptotic pathways, CDK5RAP3 knockdown MEFs cell model was generated using an inducible 4-OHT system (8 μM). Following 72 h of 4-OHT exposure, MEFs exhibited a marked reduction in CDK5RAP3 protein expression (*p* < 0.01) (Figure 7A). Flow cytometric analysis using Annexin V/PI double staining further demonstrated an increased proportion of apoptotic cells in the 4-OHT-treated MEFs (*p* < 0.05) (Figure 4B). 4-OHT treatment also enhanced NLRP3 inflammasome protein expression, along with an elevated BAX and CHOP expression (*p* < 0.05). Collectively, these results indicate that CDK5RAP3 deletion promotes inflammatory activation and apoptosis in MEFs, suggesting that CDK5RAP3 deficiency may exacerbate liver injury by synergistically amplifying these pathogenic pathway.

## 4. Discussion

The liver is an essential metabolic and detoxification organ, and hepatocellular injury represents a fundamental pathological process underlying diverse liver diseases. Accumulation of endogenous metabolic byproducts or exposure to exogenous hepatotoxins can disrupt hepatic structure and function. Liver disease has a male bias, and differences in sex hormone levels and specific gene expression are considered the main differences in the pathogenesis of liver disease, with liver cell carcinoma (HCC) being more common in men than in women [13,14]. In the acute liver injury model induced by CCl_4_ in rats, the fibrotic response of female livers to CCl_4_ was significantly weaker than that of male livers, and estradiol played a key role in inhibiting the induction of liver fibrosis in this process [15]. Together with previous reports, these observations support the concept that sex-dependent factors shape hepatic vulnerability to toxic injury, although the precise molecular mediators remain incompletely defined [16].

In this study, serum AST, ALT, and DBIL levels were significantly elevated following CCl_4_ administration, with higher levels observed in male mice than in female mice. In contrast, plasma ALB levels did not show significant differences between control and CCl_4_-treated groups, suggesting that hepatic synthetic function may be relatively preserved in this acute injury model. Moreover, TBIL levels in female mice were not markedly elevated compared with males, which is consistent with the overall milder degree of liver injury observed in female mice. Histopathological analyses further demonstrated more extensive necrosis and hepatocellular vacuolation in male mice. Rather than reiterating individual measurements, these findings collectively align with prior studies indicating that male livers are more susceptible to toxin-induced acute injury, particularly in the context of heightened inflammatory and stress responses [17].

CDK5RAP3 is a substrate adapter for the UFM1 system, and its deficiency can induce endoplasmic reticulum stress-unfolded protein response, leading to abnormal lipid accumulation, hepatic inflammation, and apoptosis [18]. As well, the downregulation or ablation of CDK5RAP3 can activate the NF-κB/NLRP3 inflammasome pathway, causing an inflammatory response, ultimately leading to cell death in udder [19]. In the present study, both CDK5RAP3 mRNA and protein levels were significantly decreased in CCl_4_-induced acute liver injury, with a more pronounced decrease observed in male mice. These data suggest that CDK5RAP3 may act as a stress-responsive regulator whose downregulation is associated with enhanced hepatic vulnerability, rather than serving as a sole determinant of injury severity. This interpretation provides biological plausibility for a link between CDK5RAP3 dysregulation and sex-dependent differences in liver injury.

Notably, CDK5RAP3 is a component of the UFL1 ligase complex within the UFM1 conjugation system, which has been reported to mediate UFMylation of several substrates, including the estrogen receptor α (ERα) co-activator ASC1 [5]. This previously described UFM1–ASC1 axis offers a plausible molecular interface through which CDK5RAP3- associated pathways may intersect with ERα signaling. Although sex hormones were not directly assessed in this study, existing evidence suggests that estrogen signaling can modulate hepatic inflammatory and stress responses [20,21], raising the possibility that hormonal regulation may interact with CDK5RAP3-associated pathways to influence sex-dependent susceptibility to liver injury.

The process of organ damage involves the participation of inflammatory responses [22]. CCl_4_-induced acute liver injury is closely related to the secretion of pro-inflammatory cytokines and inflammation [23]. In this study, the expression of pro-inflammatory cytokines IL-1β and TNF-α was significantly upregulated in the liver tissue of the model group mice, with higher levels detected in male mice. NLRP3 inflammasome activation was also enhanced, particularly in males. These observations are consistent with previous studies demonstrating sex differences in inflammatory signaling intensity and suggest that exaggerated innate immune activation may contribute to the increased injury severity observed in males [24].

Endoplasmic reticulum stress (ER stress) is one of the hallmarks of inflammation and is involved in a wide range of disease processes. Sustained or excessive cellular stress responses are often accompanied by activation of apoptotic pathways, contributing to the elimination of damaged cells [25,26]. Sustained or excessive cellular stress responses are often accompanied by activation of apoptosis-associated pathways. In this study, hepatocyte apoptosis was increased following CCl_4_ exposure, with higher apoptotic indices observed in male mice. CHOP and BAX, which are commonly associated with stress-related apoptotic signaling [27,28], were upregulated in the model group, particularly in males. These findings suggest that ER stress-associated apoptotic pathways may contribute to sex-dependent differences in liver injury severity, while acknowledging that CHOP and BAX represent downstream markers rather than comprehensive indicators of unfolded protein response activation

Several limitations of this study should be acknowledged. First, the relatively small animal sample size may limit statistical power and generalizability, although two-way ANOVA was applied to account for treatment and sex effects. Second, while inflammatory changes were suggested by histology and cytokine expression, immune cell infiltration was not directly assessed, precluding conclusions regarding the contribution of specific immune cell populations. Third, ER stress analyses focused primarily on apoptosis-associated markers without systematic evaluation of upstream UPR signaling pathways, such as PERK, IRE1, or ATF6 [29]. Accordingly, ER stress involvement should be interpreted as associative rather than definitive.

At present, it remains unclear whether CDK5RAP3 downregulation functions as an upstream driver of ER stress or occurs as a downstream consequence of stress activation during CCl_4_-induced acute liver injury. Reduced CDK5RAP3 expression was consistently associated with enhanced inflammatory and apoptotic signaling, particularly in male mice, but these observations do not establish a unidirectional causal pathway. It is plausible that CDK5RAP3 participates in a stress-responsive feedback network that modulates injury progression rather than acting as a single initiating factor.

Mechanistic insights derived from the CDK5RAP3 knockdown MEFs model should be interpreted with caution, as MEFs do not fully recapitulate hepatocyte-specific biology or the complex hepatic microenvironment. Accordingly, the in vitro findings are best viewed as complementary and supportive of the in vivo observations, rather than conclusive evidence of hepatocyte-specific mechanisms. Future studies employing hepatocyte-targeted genetic models, primary hepatocytes, immune cell-specific analyses, temporal intervention strategies, and larger animal cohorts will be required to clarify the causal relationship between CDK5RAP3 and ER stress signaling and to further elucidate the mechanisms underlying sex-dependent susceptibility to acute liver injury.

## 5. Conclusions

**Figure 8:**
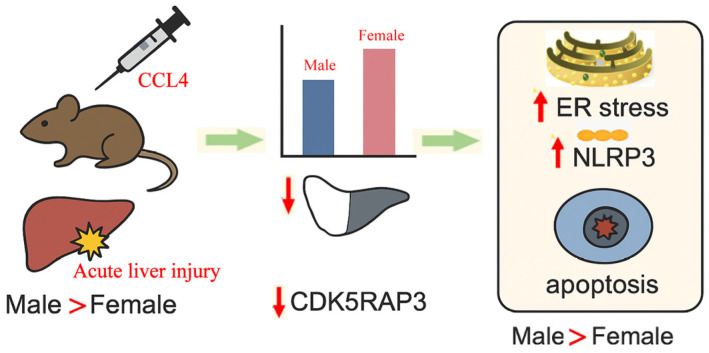
Possible mechanisms of action of CDK5RAP3 in CCL4 acute liver injury in mice of different sexes.

In conclusion, this study demonstrates clear sex differences in the susceptibility and response to CCl_4_-induced acute liver injury in mice. Reduced CDK5RAP3 expression was consistently observed in the injury model and was more pronounced in male mice, coinciding with enhanced inflammatory signaling, increased expression of ER stress-associated apoptotic markers, and more severe hepatic damage. These findings indicate that CDK5RAP3 downregulation is closely associated with stress-related and inflammatory responses during acute liver injury and may contribute to the observed sex-dependent differences in hepatic vulnerability (Figure 8). Our findings provide a framework for understanding how sex-dependent factors may intersect with cellular stress pathways in acute liver injury and highlight CDK5RAP3 as a potential stress-responsive regulator warranting further investigation.

## Figures and Tables

**Table 1:** Primers for qRT-PCR.

Gene Name	Sence Primers (5′-3′)	Anti-Sence Primers
CDK5RAP3	CTCTGACTCTTCTGGAATACCC	CTCTACTGCTCTCTGAGACAAG
TNF-α	ATGTCTCAGCCTCTTCTCATTC	GCTTGTCACTCGAATTTTGAGA
IL-1β	TCGCAGCAGCACATCAACAAGAG	AGGTCCACGGGAAAGCACAGG
Chop	ACTTGGGGACCACCTATTCCT	ATCGCCAATCAGACGCTCC
Bax	TTGCCCTCTTCTACTTTGCTAG	CCATGATGGTTCTGATCAGCTC
β-Actin	AGTACTTGCGCTCAGGAGGA	GACCTCTATGCCAACACAGT

## Data Availability

The data that support the findings of this study are available from the corresponding author upon reasonable request.

## Supplementary Materials

The following supporting information can be downloaded at https://www.mdpi.com/article/10.3390/genes17010073/s1, Table S1. Summary of all CDK5RAP3 interacting proteins in liver.

## Abbreviations

The following abbreviations are used in this manuscript:

CDK5RAP3	CDK5 regulatory subunit-associated protein 3
MEFs	mouse embryonic fibroblasts
ER	endoplasmic reticulum
CHOP	C/EBP homologous protein
